# Supplementary material for: Elderly-friendly indoor vertical dimensional layout method based on joint mobility
Source: PLoS One. 2023 May 18;18(5):e0285741. doi: 10.1371/journal.pone.0285741 (PMC10194952; doi:10.1371/journal.pone.0285741)
Supplement: Attachment — Submitted filename: Response to Reviewers.docx [file pone.0285741.s002.docx]

PONE-D-22-33201
Elderly-friendly indoor Vertical Dimensional Layout Method based on Joint Mobility
PLOS ONE

Dear Monika Błaszczyszyn,

Thank you for giving us the opportunity to submit a revised draft of the manuscript “Elderly-friendly indoor Vertical Dimensional Layout Method based on Joint Mobility” for publication in the Journal of PLOS ONE. We appreciate the time and effort that you and the reviewers dedicated to providing feedback on our manuscript and are grateful for the insightful comments on and valuable improvements to our paper.

We have revised the manuscript and answered reviewer's questions/comments with point by point hereunder according to the requirements in your letter. Hence, we are re-submitting herewith the revised manuscript.

To easily distinguish my answers from reviewer's questions/comments, we highlighted all of our answers in blue while keeping your letter and reviewers' questions/comments in black in the Response letter.

If there are any other modifications we could make, we would like very much to modify them and we really appreciate your help. We hope that our manuscript could be considered for publication in your journal. Thank you very much for your help.

Sincerely yours,

Feng Wang
Corresponding author: 
Name: Liu Jie
E-mail: [20188528@qlnu.edu.cn](mailto:20188528@qlnu.edu.cn)

**Referee Comments to Author**

**Reviewer #1:**

1. There are many grammatical mistakes and typos that should be revised carefully.

***Reply:*** Thank you very much for your comments. We have asked Editing Services to revise the manuscript completely, as shown in Fig 1. In this way, we hope our re-submitting manuscript will no longer have language problems.

**Fig 1. English editing certificate:**

2. The abstract is not structured, and should be following subheadings, like introduction, etc.

***Reply:*** We sincerely appreciate your insightful suggestions and have structured the abstract in accordance with your suggestions. This expresses the principal points of the article with enhanced clarity.

3. The importance of the study is not described in the abstract.

***Reply:*** Thank you very much indeed for your comments. We have rewritten the abstract section to emphasize the significance of the study. Please kindly review the revised abstract.

Introduction: The vertical dimensional arrangement of space is primarily influenced by the reachable range of human fingertips. Currently, this dimensional layout analysis concentrates solely on static body dimensions based on Farley's principle. However, the joint mobility of the elderly population has diminished, necessitating the identification of the factors influencing the age-friendly spatial vertical dimension layout through experimentation.

Methods: A random sample of 62 adults and 62 elderly individuals were selected to measure the joint mobility of 8 groups with varying comfort levels. The measurement results were analyzed using an independent sample t-test with SPSS software.

Results: The joint mobility of the elderly group exhibited a notable difference from that of the adult group across distinct comfort gradients. A significant reduction in the range of motion of all joints in the elderly was observed. The findings suggest that the reachable range of the upper limbs of the elderly should be considered in conjunction with joint mobility. We propose a vertical dimensional layout method for residential spaces that accommodates the joint mobility of elderly users.

Conclusion: The joint mobility of the elderly is significantly deteriorating, and the traditional vertical spatial layout method cannot adequately support the daily life of the elderly. It is essential to integrate the joint mobility factor into the vertical dimensional layout design process. In this paper, we propose an elderly-friendly vertical spatial dimension layout method. It offers a reference for subsequent planning of elderly-friendly vertical dimension layout.

4. The statistical analysis is not well reported, moreover, the statistical software is not mentioned.

***Reply:*** We sincerely appreciate your insightful suggestion. We have added to this section based on your suggestions.The statistical independent variable was age (60 years and older, and below 60 years), and the dependent variable was the mobility of the 8 joints. Differences between adults and older adults were examined in the elderly and adult cohorts of the study population. Independent samples t-test was utilized to assess the dimensional disparities between older adults and adults. SPSS 26 software was used to analyze all data, with confidence level set to 0.05.

In this study, the differences in the performance of joint movement angles between the elderly group and the adult group in the four distinct joint parts of the elbow joint, shoulder joint, trunk forward flexion, and knee joint in comfortable and effortful states were analyzed using an independent t-test research methodology. The statistical test results demonstrated a significant difference in the performance of the elderly group and the adult group at the 0.01 level in the mild grade of the elbow joint (p<0.01). In combination with the mean results, the mild grade angle of the elbow joint was 82.68° in the elderly group and 33.16° in the adult group；The statistical test results demonstrated a significant difference in the performance of the elderly group and the adult group at the 0.01 level in the mild grade of the shoulder joint (p<0.01). In combination with the mean results, the mild grade angle of the shoulder joint was

119.58° in the elderly group and 157.85° in the adult group；The statistical test results demonstrated a significant difference in the performance of the elderly group and the adult group at the 0.01 level in the mild grade of the torso forward bending angle (p<0.01). In combination with the mean results, the mild grade angle of the the torso forward bending angle was 31.82° in the elderly group and 58.19° in the adult group；The statistical test results demonstrated a significant difference in the performance of the elderly group and the adult group at the 0.01 level in the mild grade of the knee joint (p<0.01). In combination with the mean results, the mild grade angle of the knee joint was 139.95° in the elderly group and 117.47° in the adult group；The statistical test results demonstrated a significant difference in the performance of the elderly group and the adult group at the 0.01 level in the sever grade of the elbow joint (p<0.01). In combination with the mean results, the sever grade angle of the elbow joint was 42.08° in the elderly group and 22.47° in the adult group；The statistical test results demonstrated a significant difference in the performance of the elderly group and the adult group at the 0.01 level in the sever grade of the shoulder joint (p<0.01). In combination with the mean results, the sever grade angle of the shoulder joint was 151.42° in the elderly group and 176.52° in the adult group；The statistical test results demonstrated a significant difference in the performance of the elderly group and the adult group at the 0.01 level in the sever grade of the torso forward bending angle (p<0.01). In combination with the mean results, the sever grade angle of the torso forward bending angle was 44.02° in the elderly group and 91.82° in the adult group；The statistical test results demonstrated a significant difference in the performance of the elderly group and the adult group at the 0.01 level in the sever grade of the knee joint (p<0.01). In combination with the mean results, the sever grade angle of the knee joint was 119.13° in the elderly group and 66.21° in the adult group. In conclusion, the anthropometric results showed highly significant differences in joint mobility in all dimensions in the elderly group compared to the adult group (*p <* 0.01).

**Table 2. The eight joint mobility measures in study subjects by age.**

| **Dimensions** | **Group** | **Mean** | **SD** | **t** | **Sig. (2-tailed)** |
| --- | --- | --- | --- | --- | --- |
| Elbow joint angle – Mild grade | Seniors (n = 62) | 82.68 | 21.702 | 17.209 | *p* < 0.001 |
|  | Adults (n = 62) | 33.16 | 6.506 |  |  |
| Shoulder joint angle – Mild grade | Seniors (n = 62) | 119.58 | 19.432 | -12.914 | *p* < 0.001 |
|  | Adults (n = 62) | 157.85 | 12.922 |  |  |
| Torso forward bending angle – Mild grade | Seniors (n = 62) | 31.82 | 5.228 | -23.1 | *p* < 0.001 |
|  | Adults (n = 62) | 58.19 | 7.312 |  |  |
| Knee angle – Mild grade | Seniors (n = 62) | 139.95 | 9.862 | 11.719 | *p* < 0.001 |
|  | Adults (n = 62) | 117.47 | 11.444 |  |  |
| Elbow joint angle – Sever grade | Seniors (n = 62) | 42.08 | 10.053 | 12.755 | *p* < 0.001 |
|  | Adults (n = 62) | 22.47 | 6.748 |  |  |
| Shoulder joint angle –Sever grade | Seniors (n = 62) | 151.42 | 10.213 | -17.622 | *p* < 0.001 |
|  | Adults (n = 62) | 176.52 | 4.63 |  |  |
| Torso forward bending angle – Sever grade | Seniors (n = 62) | 44.02 | 7.504 | -30.044 | *p* < 0.001 |
|  | Adults (n = 62) | 91.82 | 10.034 |  |  |
| Knee angle – Sever grade | Seniors (n = 62) | 119.13 | 9.755 | 21.36 | *p* < 0.001 |
|  | Adults (n = 62) | 66.21 | 16.893 |  |  |

5. The most important point of the findings is not well-emphasized. What was the final finding and novelty of the study? It should be discussed.

***Reply:*** Thank you very much indeed for your comments. Based on your suggestion, we focus our research findings for discussion. Through experimental and statistical analysis, we found a significant decline in joint mobility in the elderly compared to adults, a phenomenon that leads to a significant reduction in fingertip reach in the elderly. In the process of vertical dimensional fingertip reach calculations for the elderly, joint mobility is a factor that cannot be ignored.

**Fig 2. Schematic diagram of Farley's principle**

The conventional vertical dimensional working area height analysis is based on Farley's principle. As shown in the Fig 2, the middle point represents the shoulder peak position; the small circle represents the normal vertical dimensional working area when the person is upright; the large circle represents the maximum vertical plane working area when the person is upright. Because people in the vertical plane can take different body postures such as upright, upper limb bending, squatting, kneeling, single leg kneeling, etc., so the vertical plane working area can be extended down to the ground. It can be seen that this principle only considers static body dimensions. Applying this principle to the vertical dimension related to the operating plane height analysis is to consider only the impact of the static physiological dimensions of the human body such as shoulder peak height and limb length. Such an analytical calculation process acquiesces to the normal mobility of all joints in the human body and is applicable for the average adult.

However, we note that some studies have demonstrated some reduction in joint range of motion in older adults. Stiffening strategy was used in the ankle joint to compensate for the degradation of the postural control system in order to promote body stability and reduce the risk of falling off. Such a strategy reduced the range of motion of the ankle joint in the elderly group [^[1]^]. Inspired by this, we finally selected four joints that were most closely related to the reachable height of the vertical dimension. The ultimate joint range of motion angles were measured and compared between the elderly group and the adult group for each joint at different comfort gradients, respectively.

It was found that the range of motion of the joints at each comfort level was significantly reduced in the elderly compared to the adults. This phenomenon resulted in a significant reduction in the reachable range of the fingertips of the elderly. In the process of calculating the height of each reference level in the vertical dimension of the elderly-friendly space, in addition to the static body size factor, the joint range of motion should be included in the calculation.

Finally, we proposed a model for calculating the reachable range of the upper limbs in the spatial vertical dimension for the elderly. The model incorporates the influence of joint range of motion in addition to static body dimensions. It is a more accurate and realistic reflection of the actual reachable height of the upper limbs of the elderly.

6. How did the authors estimate the sample sized and haw was the sampling method.

***Reply:*** Thank you very much indeed for your comments. Based on your suggestion, we have incorporated the sample size and sampling method into our paper.

The sample population was consisting of adults and the elderly. The sample size was determined based on the studies by Nurul Shahida et al [^[2]^]. and Hu et al [^[3]^]. The formula given in Annex A of ISO 15535: 2003 “General requirements for establishing anthropometric databases” was used to estimate the minimum sample size in this study. The 95% confidence interval was used for the 5th and 95th percentiles since in most cases, the anthropometric data that are of interest to product designers are those at the 5th and 95th percentiles.

n≥（3.006×CV/α）²

In this formula, n, CV and a represents the sample size, coefficient of variation and percentage of the desired relative accuracy, respectively. It was found that the minimum sample size for this study is 112(56 people in each age group). The sample size was determined based on the assumption that a relative accuracy of 10% is sufficient for the 5th and 95th percentiles and an empirical CV value of 25.Therefore, a total of 124 subjects (62 older adults aged 60 years and older versus 62 adults aged 18 to 60 years) would be sufficient for this study.

In this study, a stratified integer sampling design was employed for data extraction, ensuring representation from different constituencies. Stratified sampling was utilized to enhance the precision of the same sample size, while integer sampling was employed to improve both sample size and precision within a specified cost.

7. The baseline and demographic data of groups are not mentioned.

***Reply:*** Thank you very much indeed for your comments. The present investigation was conducted in Shandong Province. All subjects were in good health and were able to perform most of their home activity behaviors relatively smoothly and independently. The subjects had well functioning joints in all parts of the body, and no significant pathology was found. There was no history of surgery on any of the joints, and no artificial joints were replaced. Those who were unable to stand independently were excluded from the study. The mean age of the younger group was 69.3 years, and the elderly group's mean age was 41.4 years.

Prior to the start of data collection, subjects were informed that the study would develop an anthropometric database to improve the design of the vertical dimensional layout of the elderly home environment for the elderly. The measurement procedure was explained to them in detail as well. After they fully understood the purpose and procedures of the study, they could decide whether to participate in the survey or not.

8. Obviously, the orthopedic status of the elderly would be worse than the younger group, what was the novel findings of the study? They need to be reported and discussed.

***Reply:*** We sincerely appreciate your insightful suggestion. As ageing advances, more seniors are choosing to live independently. Better support from the living environment is needed. The vertical dimension layout is the biggest constraint point that affects the independent living of the elderly[^[4]^][^[5]^]. Currently, the traditional layout method for this dimension refers to the Farai principle. This method only measures the static body dimensions of adults, and then calculates the reach of the upper limbs of the human body in the vertical dimension. For the average adult, such a method is clearly adequate. However, there is no research to study and improve the layout method in the vertical dimension of space for a series of changes brought about by the physical decline of the elderly.

We invited 124 participants (of which 62 people under 60 years old constituted the adult group and 62 people 60 years old and above constituted the elderly group) to measure the maximum angle of movement of joints at different comfort levels. Four joints, which are most closely related to the attainable height of the human upper limb, were selected for measurement. The results showed that the range of motion of all joints in the elderly group was significantly reduced at different comfort levels compared to the adult group. This resulted in a consequent reduction in the reachable range of the upper extremity in the vertical dimension for the elderly. Therefore, joint mobility needs to be incorporated into the calculation factors during the design of the vertical dimension of the elderly-friendly space layout.

Finally, we propose a computational model of the reachable range of the upper limbs in the vertical dimension of space for the elderly. This calculation model takes into account not only the static body dimensions but also the joint mobility into the calculation factors. The calculation results can more accurately and realistically reflect the reachable height of the upper limbs of the elderly. The vertical dimension accessibility calculation model constructed in this study can provide a reliable basis for the vertical dimension layout of indoor space for the elderly. The elderly possess distinct characteristics when distinguishing mild and sever grades limits [^[6]^].The study also proposes a vertical division system for indoor space, which divides the indoor space into 11 reference layers according to the physical activity ability of the elderly. In addition, the study proposes the use of a comfort zone system to improve the safety and comfort of elderly living environments. Overall, the study provides a valuable reference for the design of elderly-friendly indoor space.

**Reviewer #2:**

“ I would expect the authors to indicate the novelty of the presented approaches”

***Reply:*** Thank you very much indeed for your comments. Based on your suggestion, we have rewritten the novelty of the proposed method, and we hope that this description will give you a clearer understanding of our content.

The scope of this study addresses the approach to the layout of the vertical dimension of an age-friendly space. The vertical dimensional layout is mainly influenced by the reachable height of the human upper limbs. The study targets the elderly population. Through experiments and analysis, data on physical behavior ability of the elderly group were obtained, and the calculation factor (joint mobility) of human upper limb reachable height of the elderly was increased. The accuracy of the reachable range of the upper limbs of the elderly was improved, and then an elderly-friendly spatial vertical dimension layout method was proposed.

The current reachable height calculation is based on the Farai principle, as shown in the figure, and is based on static body dimensions. Such a method is obviously sufficient for ordinary adults. However, a more targeted optimization and improvement is needed for the elderly population with a series of changes brought about by the decline of physical functions. We invited 124 participants (62 people under 60 years of age constituted the adult group and 62 people 60 years of age and older constituted the elderly group) to measure the maximum angle of motion of the joint at different comfort levels. Four joints, which are most closely related to the attainable height of the human upper limb, were selected for measurement. The results showed that the range of motion of all joints in the elderly group was significantly reduced at different comfort levels compared to the adult group. This resulted in a consequent reduction in the reachable range of the upper extremity in the vertical dimension for the elderly. Therefore, joint mobility needs to be included as a factor in the calculation during the design process of the vertical dimension of the elderly-friendly space layout. Such a calculation process will more accurately reflect the actual upper limb reachable height of the elderly.

Meanwhile, for the measurement of joint mobility angle, we not only measure the mobility angle in the effort limit state, but we also add the angle measurement in the neutral and comfortable state. The attainable height of the upper limb is calculated for each joint at different comfort grades. Each height range produces a correspondingly different level of comfort and effort zones. In conjunction with related studies, non-neutral postures such as bending and stretching, such as squatting and trunk flexion, are avoided in the home behavior of the elderly [4-5]. Six comfort zones were generated for each final zone according to the comfort level from high to low. The vertical dimensional height reference is provided for each spatial inclusion in conjunction with the use function of the specific inclusions.

1. "This study vertically divides indoor space into 11 reference layers according to the physical activity ability of the elderly users" -> in the text, I found no justification for such a division. Where does this number come from?
   ***Reply:*** Thank you very much indeed for your comments. Since the previous presentation was not clear enough, we redescribed the generation of each reference layer. to make the expression of this part clearer. According to the angle values corresponding to the three levels collected from each joint, each contact dimension is substituted into the vertical dimension reachable height calculation model to establish eight vertical reference layers of residential space for the contact dimension capability of elderly users.

**Fig 3. Eight reachable references of the upper limb at different joint comfort grades**

As shown in the Fig 3, reference layers will be generated. Also, the three static physiological dimensions of the body, namely height, eye height and shoulder height, are selected to be closely related to the vertical layout, e.g., height limits the minimum height of the vertical dimensional channel, eye height affects the height of inclusions with line of sight demand, shoulder height limits the minimum height of protrusions at the side interface of the traffic space, etc. Adding the height reference layer, the eye-height reference layer, and the shoulder height reference layer bring this total to 11 reference layers. Together, these constitute the vertical dimension layout system of indoor space for elderly users.

2. Line 402 ... apply this research method ... -> What exactly the authors mean by the term "this research method"?

***Reply:*** Thank you very much indeed for your comments. Our previous expression was not perfect, and we expressed this part more precisely. It really should not be the research method to be precise, but more precisely the computational model of the reachable height of the upper limbs of the elderly proposed in this thesis. Later efforts were made to increase the number of measurement samples and to substitute the collected data into the computational model proposed in this thesis. Through the statistical analysis of the calculation results, the final reference of each reference floor elevation for the vertical dimension layout of indoor space applicable to most of the elderly population is proposed.

**Editing notes:**
Once again, we sincerely express our gratitude to you for your serious work and for helping us to identify the shortcomings of the paper, and we have revised all the contents according to your reminders
Line:
68 Sixty -two -> Sixty-two
***Reply:*** 68 -> 119 Sixty -two -> Sixty-two

80 in ISO 7250—basic human -> in ISO 7250 — basic human
***Reply:*** 80 -> 141 7250—basic human -> 7250 — basic human
150 action for 10 -15s -> this provision is ambiguous
***Reply:*** 150 ->209 action for 10 -15s ->action for 15s
170 and Fig 3 -10. -> and Fig 3-10.
***Reply:*** These figures have been removed due to content changes.
274 As shown in Fig 9. -> this sentence seems to be unfinished

***Reply:*** 274 -> 347 The number of the Figure has been changed from 9 to 6, while the sentence has been rewritten as “ As shown in Fig 6, layer-TM is the accessible plane with torso bending behaviour when considering the comfort of elderly users.”

316 postures[30]. - > postures [30].
***Reply:*** 316 -> 413 postures [30].
Fig 3-6 -> descriptions on axes too small and unreadable

***Reply:*** These figures have been removed due to content changes.
Fig 13-14 -> descriptions are not very readable

***Reply:*** The serial numbers of Figures 13 and 14 have been changed to 10 and 11, while their descriptions have been changed as follows:

Fig 10. Vertical dimensional division of space in the shower area.

Fig 11. Vertical dimensional division of space in the washbasin area.

1. ***References***

   [] Błaszczyszyn M, Konieczny M, Pakosz P. Analysis of Ankle sEMG on Both Stable and Unstable Surfaces for Elderly and Young Women—A Pilot Study. Int. J. Environ. Res. Publ. Health. 2019;16(9):1544.
2. [] Shahida N, Zawiah M, Case K. The relationship between anthropometry and hand grip strength among elderly Malaysians. Int. J. Ind. Ergon. 2015;50:17-25.
3. [] Hu H, Li Z, Yan J, Wang X, Xiao, H, Duan J. Anthropometric measurement of the Chinese elderly living in the Beijing area. Int. J. Ind. Ergon. 2007;37(4):303-311.
4. [] Oakman J, Macdonald W, Wells Y. Developing a comprehensive approach to risk management of musculoskeletal disorders in non-nursing health care sector employees. Appl Ergon. 2014;45(6):1634-1640.
5. [] Kothiyal K, Tettey S. Anthropometric data of elderly people in Australia. Appl Ergon. 2000;31(3):329-332.
6. [] ZHANG N. Man-machine comfortable designing for bending operations of elderly. Journal of ZheJiang University (Engineering Science). 51(1):95-105.
